# Nutraceutical augmentation of circulating endothelial progenitor cells and hematopoietic stem cells in human subjects

**DOI:** 10.1186/1479-5876-8-34

**Published:** 2010-04-08

**Authors:** Nina A Mikirova, James A Jackson, Ron Hunninghake, Julian Kenyon, Kyle WH Chan, Cathy A Swindlehurst, Boris Minev, Amit N Patel, Michael P Murphy, Leonard Smith, Famela Ramos, Thomas E Ichim, Neil H Riordan

**Affiliations:** 1 Bio-Communications Research Institute, Wichita, Kansas, USA; 2 The Center For The Improvement Of Human Functioning International, Wichita, Kansas, USA; 3 The Dove Clinic for Integrated Medicine, Hampshire, UK; 4 Biotheryx Inc, San Diego, California, USA; 5 Novomedix, San Diego, California, USA; 6 Moores Cancer Center, University of California San Diego and Division of Neurosurgery, University of California San Diego, California, USA; 7 Department of Cardiothoracic Surgery, University of Utah, Salt Lake City, UT, USA; 8 Division of Medicine, Indiana University School of Medicine, IN, USA; 9 Medistem Inc, San Diego, California, USA; 10 Georgetown Dermatology, Washington, DC, USA; 11 Aidan Products, Chandler, Arizona, USA

## Abstract

The medical significance of circulating endothelial or hematopoietic progenitors is becoming increasing recognized. While therapeutic augmentation of circulating progenitor cells using G-CSF has resulted in promising preclinical and early clinical data for several degenerative conditions, this approach is limited by cost and inability to perform chronic administration. Stem-Kine is a food supplement that was previously reported to augment circulating EPC in a pilot study. Here we report a trial in 18 healthy volunteers administered Stem-Kine twice daily for a 2 week period. Significant increases in circulating CD133 and CD34 cells were observed at days 1, 2, 7, and 14 subsequent to initiation of administration, which correlated with increased hematopoietic progenitors as detected by the HALO assay. Augmentation of EPC numbers in circulation was detected by KDR-1/CD34 staining and colony forming assays. These data suggest Stem-Kine supplementation may be useful as a stimulator of reparative processes associated with mobilization of hematopoietic and endothelial progenitors.

## Introduction

Autologous bone marrow derived stem cell therapy has demonstrated benefit in early clinical trials for conditions such as critical limb ischemia [1,2], post infarct remodeling [3], stroke [4,5], and liver failure [6]. While original mechanisms of action were believed to be associated with transdifferentiation of progenitor cells to injured tissues, more recent data supports the notion that trophic/paracrine mechanisms may be involved. In this scenario the primary therapeutic function of the administered cells is production of growth factors/anti-apoptotic factors that accelerate tissue healing [7-9]. Unfortunately, despite our more advanced mechanistic understanding of cellular therapy, its widespread implementation is hindered by need for complex cell processing facilities that are only available at limited medical institutions. A more simplistic strategy would involve administration of agents capable of enhancing endogenous stem cell activity, or alternatively mobilizing bone marrow resident stem cells to increase concentration to an area of need.

It is known that subsequent to a variety of tissue injuries, such as myocardial infarction [10], stroke [11], and long bone fractures [12,13], endogenous stem cells are mobilized to the periphery, en route to the site of damage. The cytokines stromal derived factor (SDF-1) [10], vascular endothelial growth factor (VEGF) [14], and hepatocyte growth factor (HGF-1) [15] appear to act as homing signals generated by injured tissues for reparative cells. Given that stem cell mobilization appears to be associated with response to injury, one therapeutic approach has been to artificially augment mobilization subsequent to tissue damage by administration of mobilizing agents. In this manner the increased number of circulating stem cells are more available to respond to injury signals, hypothetically resulting in enhanced healing.

Granulocyte colony stimulating factor (G-CSF) and granulocyte-macrophage colony stimulating factor (GM-CSF) have been used in hematology for over two decades to mobilize donor hematopoietic stem cells [16,17]. These mobilizers have recently been used in non-hematological clinical trials to stimulate post-injury healing processes. For example, in a trial of post acute myocardial infarct patients, administration of G-CSF for 5 days resulted in significant inhibition of pathological remodeling and improvement in ejection fraction [18]. In the chronic injury setting, a trial of 45 patients with peripheral artery disease demonstrated improvement in vascular reactivity and walking time 12-weeks after a 2 week treatment with GM-CSF [19]. Improvements in endothelial function have also been reported in cancer patients post G-CSF mobilization [20]. Other studies have demonstrated the feasibility of stem cell mobilization as a possible therapy in diverse degenerative conditions such as liver failure [21,22] and ALS [23].

Chronic stimulation of stem cell mobilization is not possible using agents such as G-CSF, due to cost and possible adverse effects such as thrombosis which would be enhanced after long-term use [24]. Less invasive interventions have been reported to augment circulating stem cells such as smoking cessation or exercise [25,26]. In the current study we investigated whether a commercially-available nutraceutical, Stem-Kine (Aidan Products, Chandler AZ), was capable of increasing the number of circulating stem cells and progenitor cells. This proprietary food supplement is produced by fermentation of a combination of green tea, astralagus, goji berry extracts, with food-derived *lactobacillus Fermentum *together with ellagic acid, beta 1,3 glucan and vitamin D3. In a previous study we reported preliminary data on increased circulating endothelial progenitor cell (EPC) levels subsequent to administration (*Mikirova et al. Journal of Translational Medicine in press*). In the current study we sought to assess kinetics of EPC and stem cell mobilization in a larger population. Augmentation of both CD133 and CD34 cells in circulation was observed, as well as KDR-1+/CD34+ EPC capable of forming endothelial colonies. In contrast to pre-treatment levels, circulating stem/EPC cells were observed to undergo an approximate 2-fold increase as a result of daily supplementation.

## Materials and methods

### Study population and treatment

The study was conducted under Institutional Review Board Approval of The Center for Improvement of Human Health International, Wichita, Kansas, USA, IRB # 2009-02. Eighteen adults ages 20 -72 where recruited into the study after understanding and signing informed consent. Exclusion criteria included: systemic immune-compromised state, ongoing infection or disease conditions, and significant abnormalities in biochemistry or complete blood count panels. Subjects ceased any nutritional supplementation such as vitamins and minerals 4-5 days before trial initiation. Two 8 ml blood draws in heparinized Vacutainer tubes were collected by venipuncture before administration of Stem-Kine supplementation (day 0) and at days 1, 2, 7, and 14. Study participants were required to ingest two capsules of Stem-Kine in the morning and two in the evening for 14 days.

### Phenotypic assessment of circulating stem cells

Peripheral blood mononuclear cells (PBMC) were isolated by the Ficoll-Hypaque (Fisher Scientific, Portsmouth NH) method [27]. Briefly, blood samples were diluted two-fold with PBS and layered onto Ficoll-Hypaque in 50-ml conical tubes (Corning, Acton, MA). Each tube was centrifuged at 400 g for 30 min and the lymphocytes at the interface were collected. Cells were washed twice with RPMI 1640 medium containing 100 U/ml penicillin, 100 μg/ml streptomycin, and 2 mM L-glutamine, and subsequently resuspended in 100 ul (0.5 M cells per 100 ul) of buffer (PBS+0.5% BSA). Cells were stained with anti-CD45-FITC (BD Pharmingen), antihuman-KDR-PE, anti-CD34-PE (BD Pharminogen), CD133/AC133-PE (Miltenyi Biotec), or isotype controls recommended by manufacturer. Specifically, 10 ul of antibody was added per 100 ul of resuspended cells and refrigerated in the dark for 15 min (4-8) C. Cells were washed in 2 ml of PBS with 0.5% BSA and resuspended in 100 ul of buffer for analysis. Flow cytometry was performed using a Cell Lab Quant SC system (Beckman Coulter) equipped with 22 mW argon laser tuned at 488 nm, with the total number of cells counted cells being 30,000 per sample. The percentage of CD133 and CD34 positive cells was calculated based on the measured number of leukocytes (CD45-positive cells).

### Quantification of EPC based on colony forming ability

EPC cultures were performed using a modification of the previously described method [28-31]. Briefly, PBMC were plated on 24-well fibronectin-coated plates in Endocult liquid medium, comprised of EndoCult basal Medium and EndoCult supplement with growth factors and 2% fetal calf serum (Stem Cell Technologies, Vancouver, Canada). Cells were plated at a concentration 1 million cells per well for 5 days. For each subject colonies were plated in triplicate. Colonies represented clusters of more than 50 cells circumscribed by spindle shaped cells and were counted by microscope. As the number of colonies depends on the number of plated cells, normalization of colony number based amount of cells plated was performed twice. The coefficient for normalization was calculated from the level of ATP for the same amount of plated cells after 5 days of plating in medium without growth factors.

### HALO hematopoietic progenitor assay

The Hematopoietic/Hemotoxicity Assay via Luminescent Output (HALO, HemoGenix, Inc) assay was performed according to the manufacturer's instructions [32]. Briefly, PBMC were plated in a methylcellulose media (HemoGenix) with and without the addition of a growth factor cocktail consisting of erythropoietin (EPO, 3 U/mL), granulocyte-macrophage-colony-stimulating factor (GM-CSF, 20 ng/mL), granulocyte colony-stimulating factor (G-CSF, 20 ng/mL), interleukin-3 (IL-3, 10 ng/mL), interleukin-6 (IL-6, 20 ng/mL), stem cell factor (SCF, 50 ng/mL), thrombopoietin (TPO, 50 ng/mL), and Flt-3 ligand (10 ng/mL). Cells were plated at a concentration of 20000 cells per well in 96 well plates. After 5 days of culture, level of cellular ATP was quantified by bio-luminescence. The ratio of average values of ATP in growth factor stimulated and not stimulated cells was calculated and compared for different periods before and after intervention.

### Statistics

Differences between the groups were assessed using the non-parametric Wilcoxon rank test and P < 0.05 was considered to indicate statistical significance.

## Results

### Stem-Kine mobilizes CD34 and CD133 Cells

**Figure 1:**
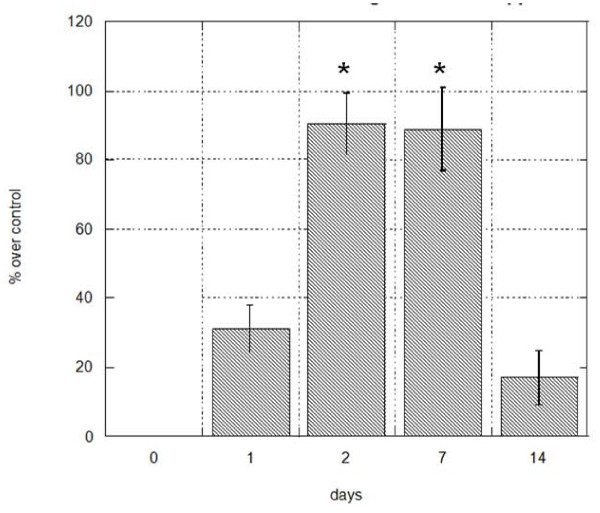
**Stem-Kine Supplementation Augments Circulating CD133 Expressing Cells**. PBMC from 18 healthy volunteers were assessed by flow cytometry for expression of CD133 at days 0, 1, 2, 7, and 14 after initiation of twice daily Stem-Kine administration. Data is presented as percentage over control of average values from all 18 subjects. *P < 0.05 compared to pre-treatment group.

**Figure 2:**
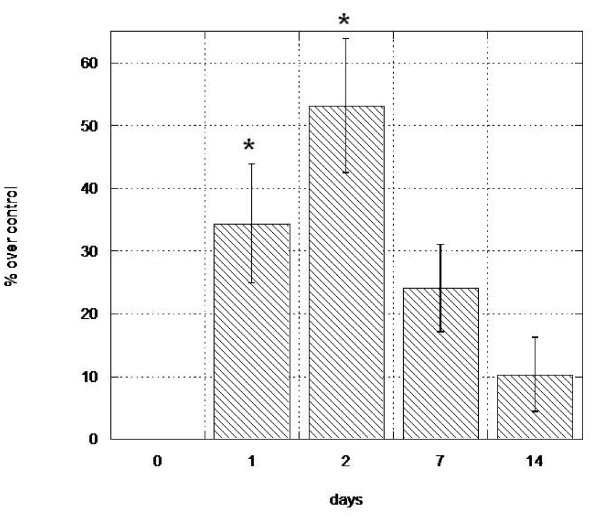
**Stem-Kine Supplementation Augments Circulating CD34 Expressing Cells**. PBMC from 18 healthy volunteers were assessed by flow cytometry for expression of CD34 at days 0, 1, 2, 7, and 14 after initiation of twice daily Stem-Kine administration. Data is presented as percentage over control of average values from all 18 subjects. *P < 0.05 compared to pre-treatment group.

Quantification of peripheral blood cells expressing the hematopoietic stem cell markers CD133 and CD34 was performed at day 0 (pre-treatment) and on days 1, 2, 7 and 14 subsequent to initiation of Stem-Kine supplementation. The average circulating CD133 cell numbers from all treated subjects peaked at 90.35% of pretreatment values (p = 0.01) on day 7 (Figure 1), whereas circulating CD34 counts reached a maximal level of 53.13% (p = .04) increase on day 2 (Figure 2). These data suggest that Stem-Kine administration is associated with significant mobilization of cells expressing hematopoietic stem cell markers. Data is presented as percentage of mononuclear cells in Additional File 1.

### Analysis of the number of the progenitor cells in circulation by HALO assay

**Figure 3:**
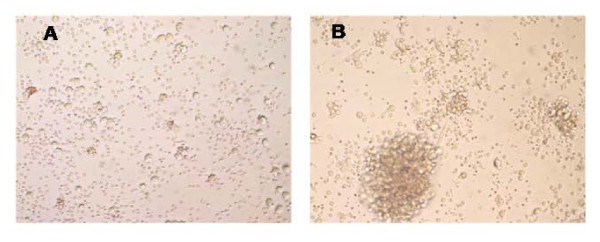
**Stimulation of Hematopoietic Progeny from PBMC (HALO Assay)**: PBMC were plated at a concentration of 20,000 cells per well and cultured on a methylcellulose matrix for 5 days supplemented with; (a) control media or (b) an optimized hematopoietic growth factor cocktail as described in Materials and Methods.

**Figure 4:**
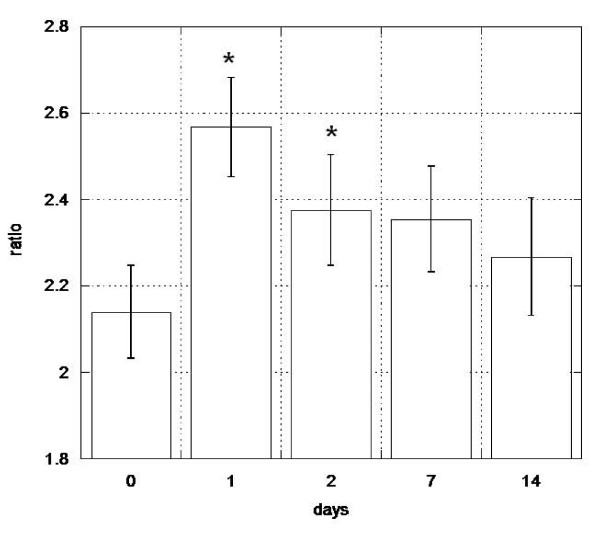
**Stem-Kine Supplementation Increases Hematopoietic Progenitor Cells in Circulation**. PBMC from subjects supplement with Stem-Kine were extracted at the indicated timepoints and cultured for 5 days in the presence of control media or hematopoietic cytokines. Ratio of ATP between activated and control cells is illustrated on the y-axis. *P < 0.05 compared to pre-treatment groups.

Cells expressing the CD34 and CD133 markers are associated with hematopoietic activity [33,34]. To assess whether Stem-Kine supplementation altered levels of functional hematopoietic progenitor cells in peripheral blood, the HALO assay [32], a modified form of the classical colony-forming assay, was used [35,36]. This technique is based on augmentation of ATP activity (indicating cellular metabolism) in cultures treated with hematopoietic growth factors versus control cultures. Increased hematopoietic cell growth was microscopically observed in treated cultures as seen in Figure 3. Data presented in Figure 4 represent the average ATP content in growth factor treated versus control (mean ± SE) for cells extracted before Stem-Kine supplementation and days 1, 2, 7, and 14. The ratio of the average ATP was increased after 24 hrs of supplementation from a pre-treatment level of 2.13 ± 0.0.44 to 2.57 ± 0.47 (p = 0.02). After 48 hrs and 7 days of supplementation, the ratio was 2.36 ± 0.5 (p = 0.05) and 2.35 ± 0.5 (p = 0.07). These data suggest Stem-Kine supplementation increases circulation of cells capable of giving rise to hematopoietic-lineage cells in vitro.

### Stem-Kine augments circulation of cells with EPC phenotype

**Figure 5:**
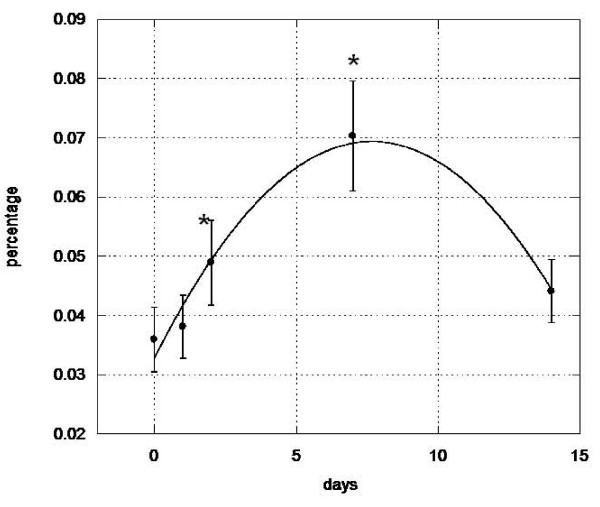
**Augmentation of KDR/CD34 positive cell numbers in circulation after Stem-Kine administration**. PBMC from 18 healthy volunteers were assessed by flow cytometry for coexpression of CD34 and KDR at days 0, 1, 2, 7, and 14 after initiation of twice daily Stem-Kine administration. *P < 0.05 compared to pre-treatment groups.

Agents such as G-CSF that induce HSC mobilization have been reported to also promote EPC mobilization [37]. Although similar molecular processes may be involved, studies suggest unique cytokine cocktails mobilize distinct stem cell populations [38,39]. Given that CD34 and CD133 are also markers of EPC [25], we sought to examine whether Stem-Kine affected EPC levels in the periphery. EPC phenotypically have been characterized by co-expression of CD34 and the kinase insert domain receptor (KDR) [40,41]. Assessment of cells bearing this phenotype was performed at similar timepoints to CD34/C133 expression pre- and post-Stem-Kine administration. Significant increases of circulating cells expressing the EPC phenotype were observed at days 2 (36.12% compared to pre-treatment control p = 0.04) and 7 (95.35% compared to pretreatment control, p = .001) as shown in Figure 5.

### Stem-Kine increases circulating cells with EPC activity

**Figure 6:**
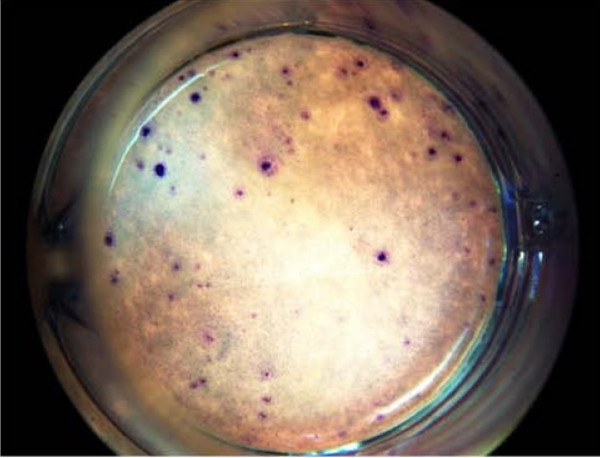
**Colony Forming Unit Endothelium Assay**: PBMC were plated on 24-well fibronectin-coated plates at a concentration of 10(6) cells per well. After 5 days of culture cells were Giemsa stained and clusters of > 50 cells were quantified as colonies.

**Figure 7:**
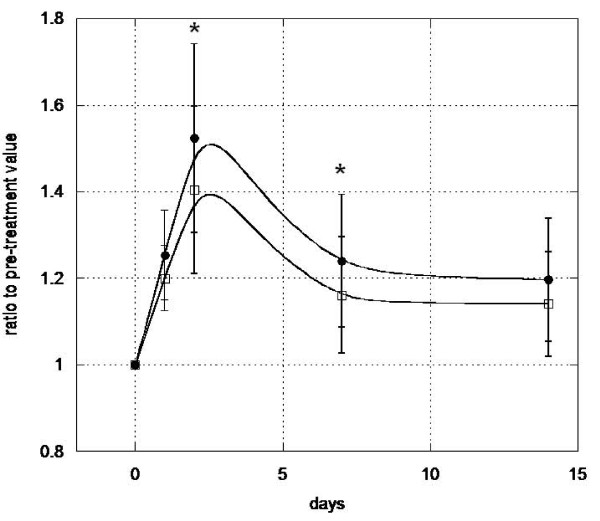
**Stem-Kine Supplementation Augments Circulating Cells with CFU-E Generating Activity**. CFU-E were generated by incubation of PBMC isolated from healthy volunteers with EndoCult Media. Data is presented as ratio to pre-treatment values. Open squares represent quantification by Alpha-Ease software, whereas closed symbols indicate quantification per viewing field by microscope. *P < 0.05 compared to pre-treatment groups.

Figure 6 illustrates morphology of a typical CFU-E. As seen in Figure 7, significant (p < 0.05) increases in colony formation were observed blood extracted on days 1 and 2. This was confirmed by visual colony counting as well as using the AlphaEase image analysis system. These data suggest Stem-Kine supplementation augments circulating levels of cells that not only bear the EPC phenotype, but are capable of forming CFU-E in vitro.

## Discussion

Hematopoietic stem cells at various stages of differentiation are localized in the bone-marrow. At a basal rate low levels of stem/progenitor cells are released from their niche and circulate in the peripheral blood [42]. Initially, upregulation of peripheral blood hematopoietic stem cell numbers was believed to be limited to post-bone marrow injury conditions [43], subsequent studies have expanded this finding to situations of inflammation [44], and peripheral tissue injury [45-47]. Hematopoietic stem cells are being increasingly recognized as having diverse non-hematopoietic functions including production of angiogenic cytokines [48], and acting as an "innate" immune cell capable of rapidly differentiating into dendritic cells for protection of the host against infections [49]. Circulating EPC are derived from the same lineage as hematopoietic cells [50], and are believed to play a role in replenishing the vasculature [51-53]. Numerous conditions including Alzheimer's Disease [54], migraine headaches [55], erectile dysfunction [56], diabetes, and peripheral vascular disease are associated with decreases in circulating EPC, possibly as a result of chronic inflammatory mediators associated with these conditions [57,58]. In contrast, acute injury such as myocardial infarction [59,60] and stroke [61], are associated with upregulated levels of these cells. Given the possibility that both hematopoietic stem cells and EPC may serve as endogenous "repair cells", we sought to assess a relatively non-invasive means of modulating these cells.

Stem-Kine is a commercially available food supplement whose intake has been associated with a variety of anecdotal reports of health improvement such as increased energy levels, enhanced skin quality, resistance to infection, and accelerated post-infection recovery. We found that administration of Stem-Kine over a 2-week course was well tolerated with no adverse effects reported. Supplementation was associated with a peak increase of approximately 53% in the number of CD34 expressing cells and and a 90% increase in CD133 cells in circulation. Furthermore, a significant augmentation of cells possessing hematopoietic colony forming activity was found in PBMC by the HALO assay. The levels of mobilization associated with Stem-Kine administration are closer to conditions that can be maintained over long term use, which is not possible with currently available mobilizers. For example, G-CSF administration at a conventionally used dose, 12 micrograms/kg for 6 days, results in a 58-fold increase in granulocytic progenitors and 24-fold increase in erythroid progenitors [62], which approximately correlated with CD34 counts [63]. Maintaining such extreme levels of mobilization over a long term increases the risk of extramedullary hematopoiesis [64], bone marrow depletion [65], and thrombosis as a result of chronic leukocytosis [24]. Indeed current indications for G-CSF recommend its use be limited to no more than 7 days for purposes of mobilization [66]. The recently approved drug AMD-3100 stimulates CD34 and CFU-GM mobilization approximately half of values obtained for G-CSF alone, however has been demonstrated to synergize with G-CSF [67]. The rapid onset and extent of mobilization limits chronic administration. As with other mobilizing agents, Stem-Kine peripheralization of CD34 and CD133 cells started to drop on day 14 of administration. This may be a physiological response towards maintaining a constant level of circulating progenitor cells. Indeed it may be possible that Stem-Kine could be beneficial in conditions associated with reduced progenitor cells such as diabetes or in smokers which possess lower baseline values as compared to controls [25,26,57,58].

While we correlated an increase in hematopoietic colonies with Stem-Kine induced upregulation of peripheral blood CD34 and CD133 cells, given that these markers are also found on EPC [25], we evaluated the possibility that circulating EPC numbers were also increased. We observed maximal increases (almost doubling) of CD34+ KDR+ cells in PBMC occurring at day 7 of supplementation, whereas peak CFU-E activity occurred at day 2. The reason for this discrepancy is not known, but potentially may be related to existence of various subsets of cells with EPC potential residing outside of the CD34+ KDR+ fraction. Further studies are required to elucidate functional importance of the variable kinetics of mobilization, as well as possible differences on long-term versus short-term circulating EPC.

The mechanism of Stem-Kine mediated mobilization remains unknown. One possibility is that a temporary disruption of the SDF-1a/CXCR4 axis is occurring, in a similar manner to mobilization induced by G-CSF or cyclophosphamide [68]. Not mutually exclusive is the possibility that Stem-Kine is activating bone marrow resident macrophages, elaborating cytokines associated with mobilization [69]. We are favoring this possibility based on agents that induce mobilization in the relative potency range associated with Stem-Kine. For example, specific molecular weight ranges of hyaluronic acid have been demonstrated to induce mild mobilization [70,71], an effect that is associated with bone marrow macrophage production of IL-1 and IL-6 [72]. Peptidoglycan components which are found in Stem-Kine are known to activate macrophages and stimulate production of IL-6 [73].

To our knowledge, this is the first study demonstrating profound mobilization effect with possible clinical significance by a food supplement-based approach. The nutritional supplement StemEnhance, is an extract of the cyanobacteria Aphanizomenon flos-aquae [74]. Jensen et al which demonstrated a 25% increase in circulating CD34+ cells, which peaked at 60 minutes-post administration and subsided at 120 minutes [75]. Another nutraceutical product, Nutra-Stem, is composed of a combination of blueberries, green tea extract, carnosine, and vitamin D3. In vitro activity on proliferation of human bone marrow cells was assessed, in which a 60% enhancement of growth was reported [76]. Bone marrow cells from mice supplemented with Nutra-Stem were protected from in vitro exposure to hydrogen peroxide at up to approximately 40% [77]. These data suggest the possibility of nutritional modulation of stem cell compartments, but do not provide results on mobilization. Further research is required to assess physiological effects in humans.

In conclusion, the current study suggests feasibility of significant mobilization of cells expressing hematopoietic stem cell and EPC markers and properties. The area of nutritional modulation of the stem cell compartment offers significant benefit in treatment of a wide variety of degenerative diseases. However given commercial pressures associated with this largely unregulated field, we propose detailed scientific investigations must be made before disease-associated claims are made by the scientific community.

## Competing interests

Neil H Riordan is a shareholder of Aidan Products. All other authors have no competing interests.

## Authors' contributions

NHR and NAM designed experiments, interpreted data and conceptualized manuscript. RH, JAK, JK, KWA, CAS, BM, ANP, MPM, LS, FR, and TEI provided detailed ideas and discussions, and/or writing of the manuscript. NAM and JAJ performed the experiments. All authors read and approved the final manuscript.

## Supplementary Material

Additional file 1 **Progenitor Cell Numbers Expressed as a Percentage of Peripheral Blood Mononuclear Cells**. The data provided represent number of progenitor cells (CD133, CD34, and cells with EPC functional activity) as a percentage of peripheral blood mononuclear cells. Click here for file
