# Supplementary material for: Nutraceutical augmentation of circulating endothelial progenitor cells and hematopoietic stem cells in human subjects
Source: J Transl Med. 2010 Apr 8;8:34. doi: 10.1186/1479-5876-8-34 (PMC2862021; doi:10.1186/1479-5876-8-34)
Supplement: Additional file 1 — Progenitor Cell Numbers Expressed as a Percentage of Peripheral Blood Mononuclear Cells. The data provided represent number of progenitor cells (CD133, CD34, and cells with EPC functional activity) as a percentage of peripheral blood mononuclear cells. [file 1479-5876-8-34-S1.DOC]

|  | time points (days) | 0 | 1 | 2 | 7 | 14 |
| --- | --- | --- | --- | --- | --- | --- |
| CD133 % | average | 0.0105 | 0.0138 | 0.0201 | 0.0199 | 0.0123 |
|  | SD | 0.0044 | 0.0079 | 0.0134 | 0.0103 | 0.0053 |
|  | SE | 0.0010 | 0.0019 | 0.0032 | 0.0024 | 0.0012 |
|  | % of improvement |  | 31.0 | 90.4 | 88.8 | 16.9 |
| CD34 % | average | 0.0142 | 0.0200 | 0.0215 | 0.0185 | 0.0164 |
|  | SD | 0.0089 | 0.0192 | 0.0158 | 0.0133 | 0.0107 |
|  | SE | 0.0026 | 0.0055 | 0.0046 | 0.0038 | 0.0031 |
|  | % of improvement |  | 41.3 | 51.8 | 30.5 | 16.0 |
| EPCs % | average | 0.0360 | 0.0381 | 0.0489 | 0.0702 | 0.0441 |
|  | SD | 0.0194 | 0.0227 | 0.0303 | 0.0394 | 0.0135 |
|  | SE | 0.0046 | 0.0054 | 0.0071 | 0.0093 | 0.0032 |
|  | % of improvement |  | 6.0 | 36.1 | 95.4 | 22.6 |
| WBC (K/ul) | average | 6.4 | 7.0 | 7.3 | 6.6 | 6.5 |
|  | SD | 0.128 | 0.14 | 0.146 | 0.132 | 0.13 |
| lymphocytes (K/ul) | average | 1.8 | 2.1 | 2.1 | 2.0 | 1.8 |
|  | SD | 0.036 | 0.042 | 0.042 | 0.04 | 0.036 |

**ADDITIONAL FILE 1: Progenitor Cell Numbers Expressed as a Percentage of Peripheral Blood Mononuclear Cells**
